# Interceptive Orthodontics with Resin Turbos for Pseudo-Class III Malocclusions

**DOI:** 10.1155/2019/1909063

**Published:** 2019-05-05

**Authors:** Neal D. Kravitz

**Affiliations:** 25055 Riding Plaza, Suite 110, South Riding, , Virginia 20152 , USA

## Abstract

**Background and Overview:**

Lingual eruption of the permanent maxillary central incisors in the early mixed dentition can result in a traumatic anterior crossbite, causing mobility and gingival recession to the opposing mandibular incisors.

**Case Description:**

This case report presents a common finding of a 7-year-old boy with a dental crossbite and pseudo-Class III malocclusion caused by lingual eruption of the maxillary central incisors. An interceptive phase of orthodontic treatment was provided by bonding a beveled resin turbo on the mandibular incisors. The crossbite was corrected in 3 months without any orthodontic appliances. In the absence of the traumatic occlusion, the mandibular incisors stabilized and the gingival tissue was expected to regenerate.

**Conclusions and Practical Implications:**

Dentists and orthodontists can place beveled resin turbos on the mandibular incisors to jump an anterior dental crossbite conservatively, without the use of orthodontic brackets and wires.

## 1. Introduction

Resin *turbos* are created by bonding composite material to the palatal or occlusal surfaces of the teeth. They are commonly used by orthodontists to prevent heavy occlusal contact with lower braces in patients with deep overbites. Turbos that are beveled, however, can be used to correct mild dental crossbites. These turbos, also referred to as *functional turbos*, are constructed with beveled occluding surfaces that guide the opposing teeth towards their desired position.

**Figure 1:**
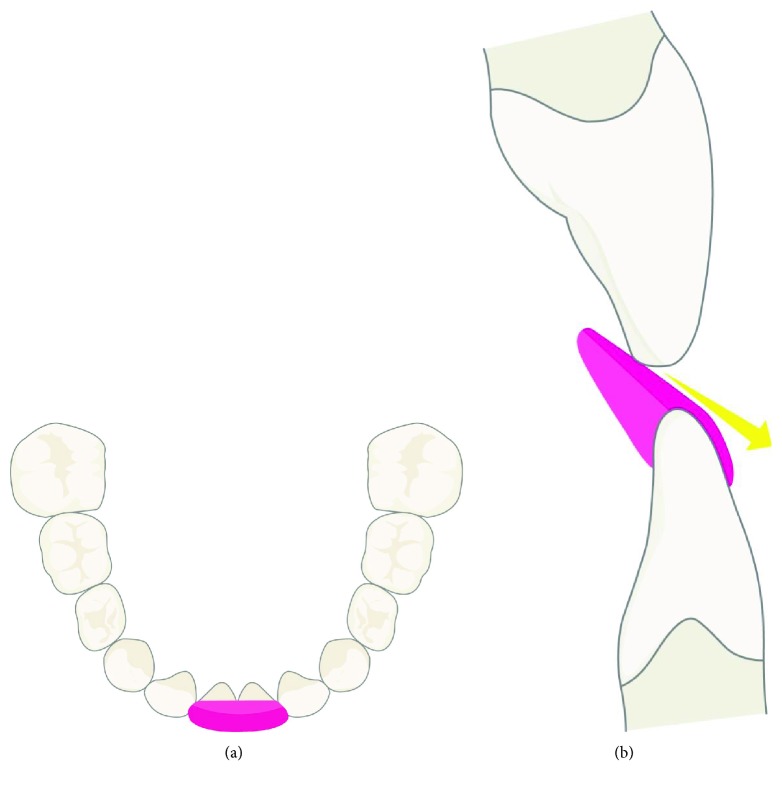
(a) Illustration of the functional turbo (pink) from the occlusal. (b) Illustration of the functional turbo from the sagittal. The turbo nudges the maxillary incisor forward and the mandible backward.

The most common use of functional turbos is for the correction of an anterior dental crossbite. In this application, resin material is bonded to the incisal edges of two or more lower incisors and then beveled lingually with a handpiece (Figures 1(a) and 1(b)). Upon contact with the beveled surface, the upper incisors are nudged forward and the lower jaw is directed posteriorly. The dental crossbite is corrected in approximately 3 months, oftentimes in the absence of orthodontic brackets and wires.

## 2. Case Report

**Figure 2:**
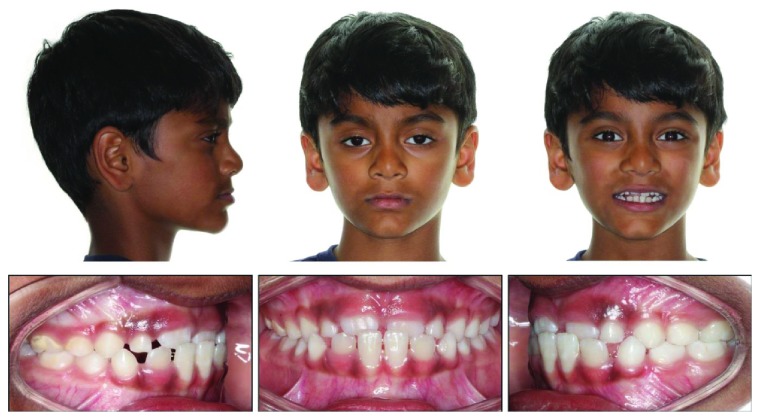
Pretreatment composite.

A 7-year-old Indian-American boy was referred by his pediatric dentist for evaluation of an anterior dental crossbite caused by lingual eruption of the maxillary central incisors (Figure 2). The traumatic occlusion had caused mobility and early gingival recession to the opposing mandibular central incisors. The patient's chief symptom was moderate tooth pain during mastication. His medical history was normal and healthy, with no family history of prognathism.

Intraoral examination revealed a pseudo-Class III malocclusion in the early mixed dentition. Both maxillary central incisors were partially erupted and positioned in an anterior crossbite to the mandibular central and left lateral incisors. The patient displayed an end-to-end incisal relationship when the mandible was positioned into centric relation (CR). The mandibular central incisors displayed 3 mm of gingival recession compared to the mandibular right lateral incisor that was not in crossbite. These teeth also exhibited +2-degree mobility and were sensitive to palpation. The extraoral examination showed a slightly concave profile, with no apparent facial asymmetry.

**Figure 3:**
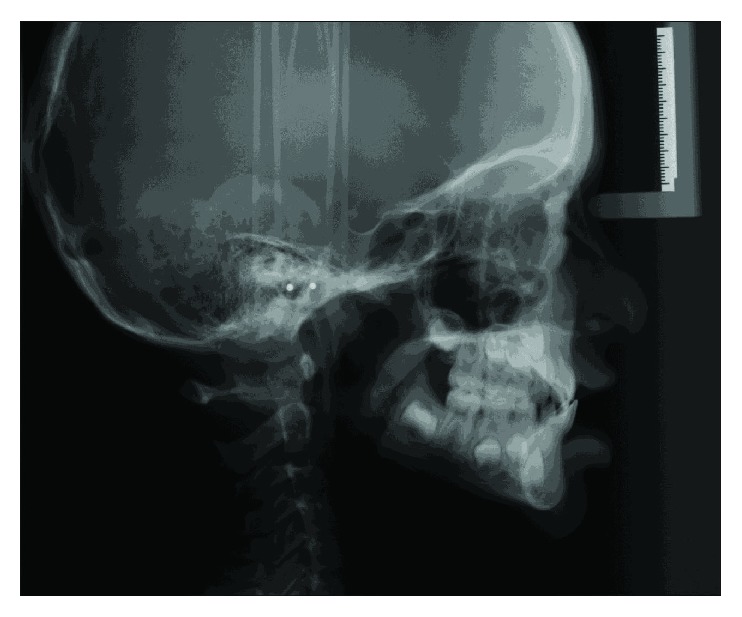
Pretreatment cephalograph.

Cephalometric analysis revealed a mild Class 3 skeletal relationship (ANB = 0°) in centric occlusion, with a short midface length (Co − A = 71.5 mm) and a normal mandibular length (Go − Gn = 67.1 mm) (Figure 3). The maxillary central incisors were retroclined (U1 − SN = 94°) while the mandibular central incisors displayed proper angulation (L1 − MP = 98°). Accordingly, the upper lip was set back too far (Upper Lip to E − Plane = −4 mm). The panoramic radiograph revealed a full complement of adult teeth.

The treatment objectives were to achieve positive overlapping of the anterior teeth and eliminate the traumatic occlusion to the mandibular incisors. The parents were informed that waiting to intervene until adolescence could result in further tooth mobility and gingival recession.

The family was presented the following 3 treatment options, ranging from the most comprehensive to conservative:

1. Upper and lower fixed partial braces on the permanent molars and incisors
2. A removable orthodontic appliance, such as an upper Hawley bite plate with a finger spring or a mandibular inclined plane
3. Bonding of a functional resin turbo in the absence of fixed or removable orthodontic appliances

The first option would enable space consolidation and detailing of the maxillary incisors, but it would have the disadvantages of increased cost and treatment duration. The family also was nervous about whether their child could cope with the discomfort of brackets and bands at his age. The second option would correct the crossbite without braces, but the treatment's success would depend on the patient's compliance in wearing the removable retainer. Therefore, the family chose the third option of bonding a functional resin turbo to provide a noncompliant alternative in the absence of braces.

**Figure 4:**
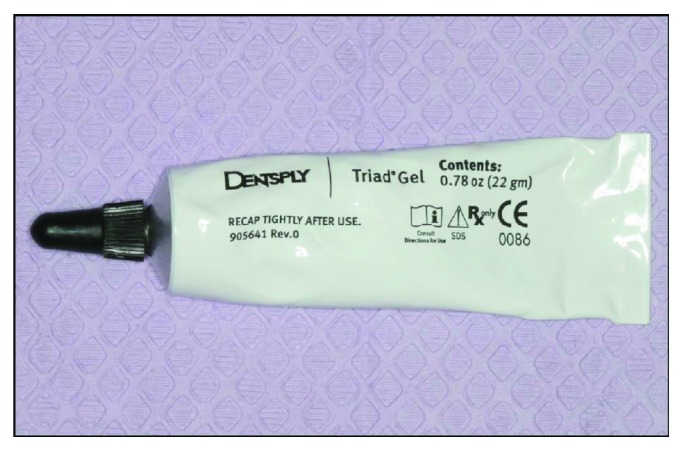
Triad® Gel tube.

The turbo material chosen was Triad® Gel, which is an acrylic resin liquid that is used traditionally to create bite plates and modify dental casts (Figure 4). It is composed primarily of methacrylate mixed with a small amount of silica glass (WT 1-10%). Triad® Gel is packaged in small tubes and comes in four assorted colors: clear colorless, clear pink, clear blue, and clear red.

**Figure 5:**
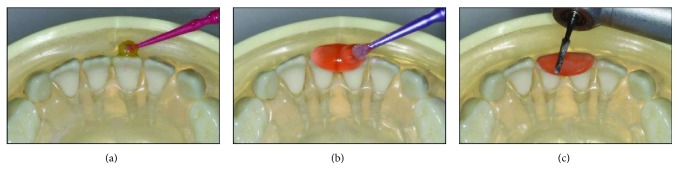
(a) Rubbing the facial and incisal surfaces with a self-etch adhesive L-Pop™. (b) Applying the Triad® Gel with a microbrush. Alternatively, it can be applied with a repurposed flowable syringe. (c) Beveling the cured material lingually with a handpiece.

**Figure 6:**
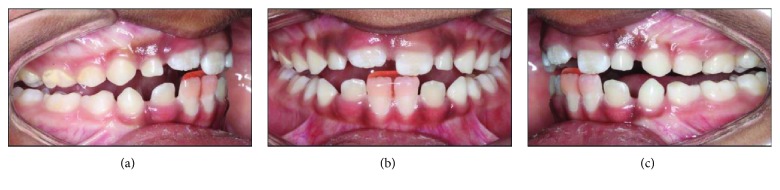
Progress records immediately after placing the functional turbo.

The Triad® Gel was bonded to the teeth with a standard etch and prime technique (Figures 5(a)–5(c)). First, the mandibular central incisors were prepared by rubbing the facial and incisal surfaces with a self-etch adhesive L-Pop™. The Triad® Gel was then applied incrementally with a microbrush and light-cured. The material was built vertically, beginning from the facial surfaces, and extended lingually. Both incisors were bonded together to provide stabilization. A handpiece was then used to create the upward sloping lingual bevel (Figure 6).

The occlusion was tested to ensure the turbo was at the appropriate height and thickness. If the material was too high, the patient would be uncomfortable while eating. Most importantly, if the material did not extend far enough lingually, the patient could simply posture his mandible forward and close fully into an underbite in centric occlusion. The family was informed that it would take several days for the patient to become accustomed to having the turbo in his mouth.

**Figure 7:**
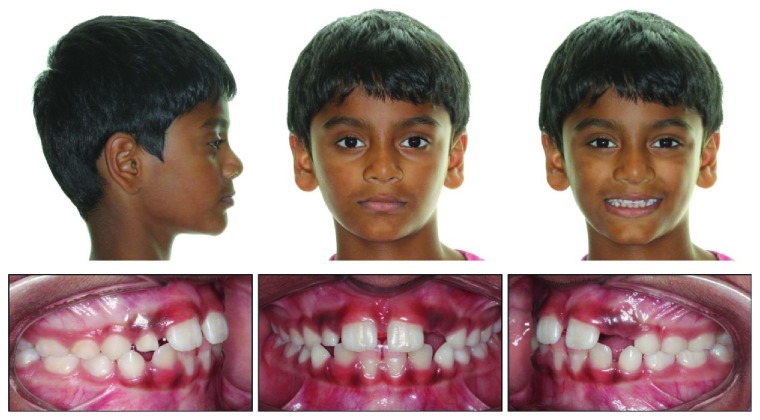
Posttreatment composite at the following visit after 3 months.

The patient returned to the office for his first checkup after 3 months, and the crossbite had fully corrected (Figure 7). The disclusion caused by the functional turbo had also allowed the maxillary central incisors to further erupt. During this appointment, the Triad® Gel was removed with a handpiece. The mandibular central incisors were no longer pathologically mobile or sensitive to palpation. Successful interceptive orthodontic treatment was completed in 1 visit, in the absence of fixed or removable orthodontic appliances.

**Figure 8:**
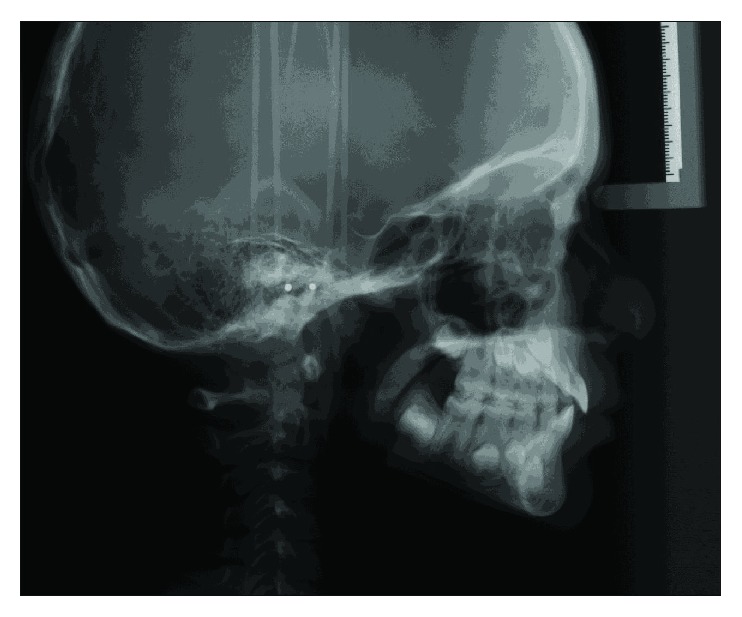
Posttreatment cephalograph.

**Figure 9:**
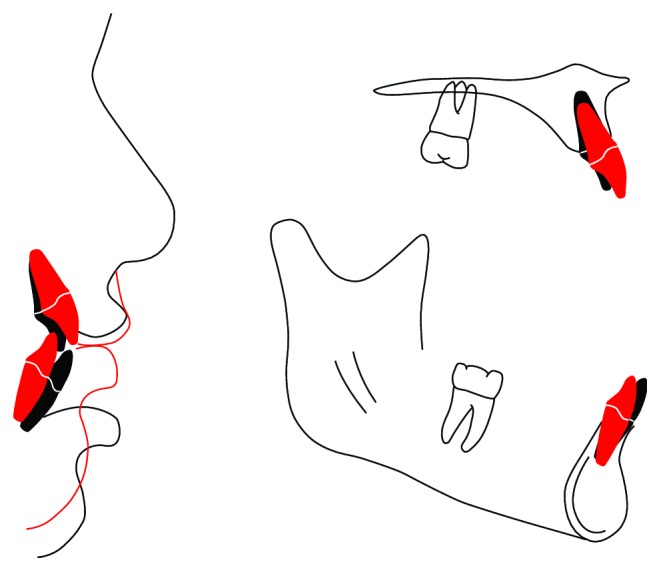
Superimposition.

At the end of the treatment, the patient achieved positive overlapping of the anterior teeth (overjet = 4 mm), and his facial profile also was less concave (Figure 8). Cephalometric superimpositions showed that the maxillary central incisors had protruded and proclined 4 mm and 9°, respectively (Figure 9). The upper lip also moved forward 3 mm, into an ideal position (Table 1). The family was pleased with the improved smile esthetics and bite closure. The patient was placed on a 6-month recall schedule to monitor the eruption of his remaining permanent teeth and the readiness for comprehensive orthodontic treatment.

## 3. Discussion

Functional resin turbos that are used for correction of anterior crossbites are most appropriate for patients with pseudo-Class III malocclusions [1, 2]. A pseudo-Class III malocclusion is not a true skeletal Class III malocclusion associated with a longer mandible; rather, it is an anterior crossbite caused by a protrusive shift of the mandible [3]. The protrusive shift occurs as a result of an occlusal interference from the retroclination of the maxillary incisors. Essentially, the forward posturing of the mandible exaggerates a skeletal discrepancy.

A pseudo-Class III malocclusion often displays the following diagnostic characteristics: (1) likely no family history of prognathism, (2) forward position of the mandible in centric occlusion, (3) end-to-end incisor relationship when the patient is guided into centric relation, (4) decreased midface length, (6) normal mandibular length, (7) retroclined upper incisors and normal lower incisors, and (8) a retrusive upper lip [4]. These malocclusions are also found primarily in the deciduous or mixed dentitions [5]. Our patient presented with all of these characteristics.

Early correction of a pseudo-Class III malocclusion is recommended to minimize periodontal complications associated with the anterior crossbite. Trauma from the occlusion and excessive masticatory forces promotes gingival recession around the affected mandibular incisors [6], as was seen with our patient. Timely orthodontic correction, however, often leads to spontaneous reversal of the gingival margin level after 1 year [7]. Furthermore, the malocclusion is unlikely to reoccur after orthodontic treatment [8].

By adding the functional turbo to the mandibular central incisors, the anterior crossbite and protrusive shift of the mandible were immediately eliminated, as the pressure from the turbo's inclined plane nudged the maxillary central incisors forward. The functional turbo also caused the mandible to rotate clockwise in an opening rotation, which helped correct the malocclusion. In the absence of the traumatic occlusion, the mandibular incisors stabilized and the gingival tissue was expected to regenerate.

Triad® Gel was selected for the functional turbo as its composition is primarily methacrylate, which poses a low risk of abrasion to the maxillary incisors. By comparison, restorative composites pose a higher risk for wear because they contain a higher percentage of abrasive glass fillers, such as quartz [1]. The primary disadvantage of Triad® Gel is the exothermic reaction that is triggered when the material is light-cured, which can cause pulpal hyperemia. Therefore, the material was applied and cured incrementally to minimize tooth discomfort [1].

Most importantly, the functional resin turbo was a minimalistic method to provide interceptive orthodontic treatment. Although braces placed on the permanent maxillary incisors are the traditional fixed method used to correct an anterior crossbite in the early mixed dentition [9], this approach has disadvantages, including the potential for decalcifications, increased treatment cost and number of appointments, and fatigue when the child is due for comprehensive treatment. With regard to interceptive orthodontics in younger patients, a streamlined approach is most favorable.

## 4. Conclusion

This case report presents a common finding of a 7-year-old boy with a pseudo-Class III malocclusion and anterior crossbite, resulting in mobility and gingival recession to the occluding mandibular incisors. A short phase of interceptive orthodontics was provided using only a functional resin turbo. The crossbite was corrected in 3 months without any orthodontic appliances. This case report highlights both the importance of early diagnosis and the effectiveness of conservative orthodontic treatment.

## Figures and Tables

**Table 1:** Cephalometric measurements.

Measurement	Pre	Post
SNA (°)	76	76
SNB (°)	76	75
ANB (°)	0	1
FMA (°)	23	24
U1-NA (mm)	2	5
U1-SN (°)	94	103
L1-NB (mm)	4	2
L1-MP (°)	98	89
Lower lip to E-plane (mm)	1	1
Upper lip to E-plane (mm)	-4	-1

Pre- and posttreatment cephalometric measurements.
